# Resilience to Loneliness Amongst Older Adults Living in the Bronx, NY, During the COVID-19 Pandemic

**DOI:** 10.1093/geroni/igaa057.3494

**Published:** 2020-12-16

**Authors:** Karra Harrington, Ruixue Zhaoyang, Stacey Scott, Jennifer Graham-Engeland, Martin Sliwinski

**Affiliations:** 1 Pennsylvania State University, University Park, Pennsylvania, United States; 2 The Pennsylvania State University, University Park, Pennsylvania, United States; 3 Stony Brook University, Stony Brook, New York, United States; 4 Penn State University, University Park, Pennsylvania, United States

## Abstract

Older adults may be particularly vulnerable to experiencing loneliness as a result of stay-at-home and social distancing orders during the COVID-19 pandemic. This study evaluated change in loneliness following the COVID-19 outbreak, using a longitudinal design and a validated loneliness measure, in a well-characterized sample who are at heightened risk for COVID-19 due to both age and location. The study included n = 226 older adults aged 70-90 years old, living in the Bronx, New York City, who had completed the 3-item Loneliness Scale prior to and during the peak of the COVID-19 outbreak in New York City. There was no evidence of significant increases in mean loneliness from pre- to post-COVID-19 outbreak. Multiple regression analyses were used to identify risk and protective factors for change in loneliness during the COVID-19 outbreak, adjusting for pre-outbreak loneliness. Living alone, higher levels of education, greater worry about contracting the coronavirus, and limiting of daily exercise activities were risk factors for greater loneliness after the outbreak. In contrast, Black race, older age, greater social support and frequent social interactions via video call, all related to lower levels of loneliness after the outbreak.The outcomes of this study demonstrate substantial resilience among older adults to loneliness in response to the COVID-19 pandemic, and highlight key risk and protective factors that may play an important role in individual differences in loneliness as pandemic-driven isolation continues.

